# Molecular mechanism of phosphoinositides' specificity for the inwardly rectifying potassium channel Kir2.2†
†Electronic supplementary information (ESI) available. See DOI: 10.1039/c8sc01284a


**DOI:** 10.1039/c8sc01284a

**Published:** 2018-09-05

**Authors:** Xuan-Yu Meng, Seung-gu Kang, Ruhong Zhou

**Affiliations:** a State Key Laboratory of Radiation Medicine and Protection , School for Radiological and Interdisciplinary Sciences (RAD-X) , Collaborative Innovation Centre of Radiation Medicine of Jiangsu Higher Education Institutions , Soochow University , Suzhou 215123 , China; b IBM Thomas J. Watson Research Center , Yorktown Heights , NY 10598 , USA . Email: ruhongz@us.ibm.com; c Department of Chemistry , Columbia University , New York , NY 10027 , USA

## Abstract

Phosphoinositides are essential signaling lipids that play a critical role in regulating ion channels, and their dysregulation often results in fatal diseases including cardiac arrhythmia and paralysis. Despite decades of intensive research, the underlying molecular mechanism of lipid agonism and specificity remains largely unknown. Here, we present a systematic study of the binding mechanism and specificity of a native agonist, phosphatidylinositol 4,5-bisphosphate (PI(4,5)P_2_) and two of its variants, PI(3,4)P_2_ and PI(3,4,5)P_3_, on inwardly rectifying potassium channel Kir2.2, using molecular dynamics simulations and free energy perturbations (FEPs). Our results demonstrate that the major driving force for the PI(4,5)P_2_ specificity on Kir2.2 comes from the highly organized salt-bridge network formed between the charged inositol head and phosphodiester linker of PI(4,5)P_2_. The unsaturated arachidonic chain is also shown to contribute to the stable binding through hydrophobic interactions with nearby Kir2.2 hydrophobic residues. Consistent with previous experimental findings, our FEP results confirmed that non-native ligands, PI(3,4)P_2_ and PI(3,4,5)P_3_, show significant loss in binding affinity as a result of the substantial shift from the native binding mode and unfavorable local solvation environment. However, surprisingly, the underlying molecular pictures for the unfavorable binding of both ligands are quite distinctive: for PI(3,4)P_2_, it is due to a direct destabilization in the bound state, whereas for PI(3,4,5)P_3_, it is due to a relative stabilization in its free state. Our findings not only provide a theoretical basis for the ligand specificity, but also generate new insights into the allosteric modulation of ligand-gated ion channels.

## Abstract 2

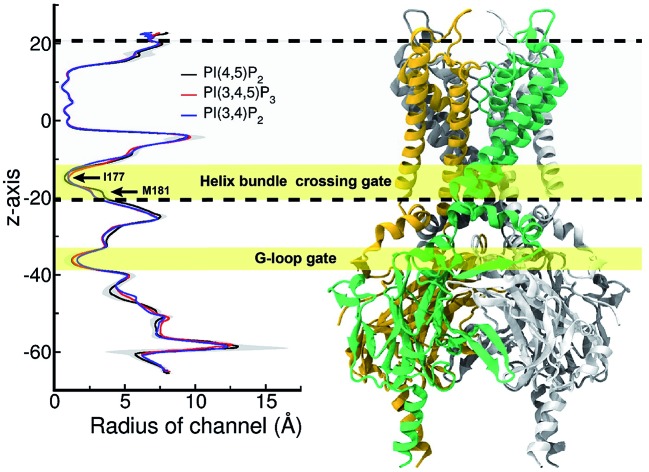
We investigated the binding mechanism of PI(4,5)P_2_ and variants on the inwardly rectifying potassium channel, Kir2.2. Our results not only demonstrated the molecular origin for their binding specificity, but also revealed the major driving forces.

## Introduction

Phosphoinositides (PIPs) are one class of signaling lipid molecules distributed broadly in the inner leaflet of the plasma membrane, which play critical roles in diverse physiological functions, such as regulating the activity of ion channels and transporters, endocytosis and exocytosis, and calcium signaling.^1^–^6^ The most abundant phosphoinositide in membranes is phosphatidylinositol 4,5-bisphosphate (PI(4,5)P_2_) whose well-known functional roles include the generation of two critical second messengers, inositol trisphosphate (IP3) and diacyl glycerol (DAG) after being hydrolyzed, as well as its broad regulation of almost all ion channels and many transporters.^7^–^11^ Structurally, PI(4,5)P_2_ is composed of an inositol head group with two phosphates on its 4′ and 5′ positions, linked by two fatty acid tails (*i.e.*, stearic and arachidonic acids) through a phosphodiester linker in-between. Variations in the number and position of phosphorylation on the inositide group produce different derivatives, such as PI(3,4)P_2_ and PI(3,4,5)P_3_.

**Fig. 1:**
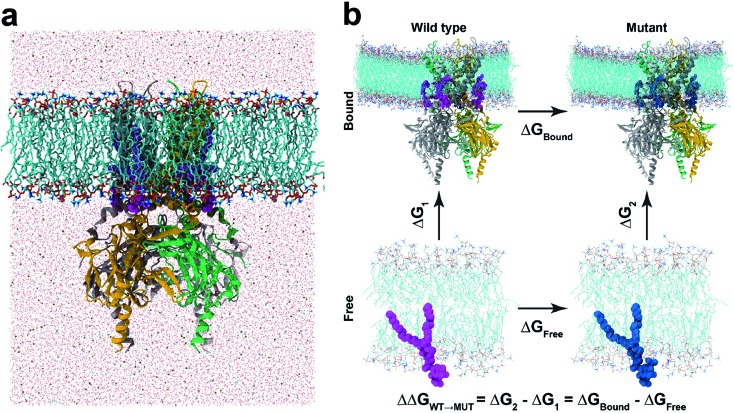
Molecular dynamics and free energy perturbation simulation. (a) Molecular dynamics set-up. KIR2.2 bound with four PI(4,5)P_2_ (marked in magenta) is embedded in a POPC membrane bilayer, as solvated with 150 mM KCl. (b) Thermodynamic cycle for free energy perturbation calculations. The binding affinities of mutant phosphoinositides (PI(3,4,5)P_3_ and PI(3,4)P_2_) are calculated relative to that of PI(4,5)P_2_ by calculating FEPs for Δ*G*_bound_ and Δ*G*_free_, where the wild-type (PI(4,5)P_2_) and mutants (PI(3,4,5)P_3_ or PI(3,4)P_2_) are depicted in magenta and blue balls, respectively.

Since the early 1990s, people realized that PI(4,5)P_2_ directly interacts with the inwardly rectifying potassium (Kir) channels and controls the channel activity.^12^–^14^ Many studies focused on uncovering the molecular mechanism of PI(4,5)P_2_–Kir interactions.^15^–^19^ The binding of PI(4,5)P_2_ upon Kir channels involves a very exquisite way: it locates at two different interfaces, with one at the vertical interface between the transmembrane and cytoplasmic domains, and the other at the horizontal interface between the adjacent subunits of the channel (more below in Fig. 1). Thus, one PI(4,5)P_2_ may interact with two domains as well as two subunits simultaneously. The co-crystal structure of the chicken Kir2.2 with PI(4,5)P_2_ indicates that PI(4,5)P_2_ may induce the helix-formation of tether-helix in one subunit and C-terminus of the Slide helix in the adjacent subunit.^18^ The main driving force for PI(4,5)P_2_ and Kir channels is the electrostatic attraction: PI(4,5)P_2_ bears negative charges under the physiological conditions and the binding site of Kir complementarily consists of multiple positive charged residues.^19^ A series of important basic residues that form the salt bridges with PI(4,5)P_2_ were also indicated through electrophysiology and crystallography studies.^17^–^19^ Mutagenesis of these basic residues either decreases the channel sensitivity to PI(4,5)P_2_ or damages the channel activity.^19^,^20^

Regarding the electrostatic interaction for the PI(4,5)P_2_–Kir channel, it is interesting to raise the question whether Kir could be activated by other phosphoinositides like PI(3,4)P_2_ and PI(3,4,5)P_3_, which have high structural and chemical similarity to the native agonist PI(4,5)P_2_. Both PI(3,4)P_2_ and PI(3,4,5)P_3_ are phospholipid components of cell membranes involved in many important signal transduction pathways. Logothetis's lab recently utilized electrophysiology techniques to measure the activity of a series of Kir channels, Kir2.1, Kir2.2, K3.4*, and Kir6.2, repetitively, under PI(4,5)P_2_, PI(3,4)P_2_ and PI(3,4,5)P_3_.^21^ They discovered that on one extreme PI(3,4)P_2_ and PI(3,4,5)P_3_ hardly activate the channel (*i.e.*, Kir2.1, a typical Kir channel that requires PI(4,5)P_2_ for maintaining normal function) (Group 1); and on the other extreme, the current levels induced by PI(4,5)P_2_, PI(3,4,5)P_3_, or PI(3,4)P_2_ were comparable (*i.e.*, Kir6.2, also sensitive to long chain acyl CoA) (Group 4). In other cases, their responses to PI(3,4)P_2_ and PI(3,4,5)P_3_ were in-between Kir2.1 and Kir6.2. For example, the mouse Kir2.2 had no response to PI(3,4)P_2_, but was partially activated by PI(3,4,5)P_3_ (its current reached about 30% of PI(4,5)P_2_'s) (Group 2). For Kir3.4*, PI(3,4)P_2_ activated the current level by 20% of PI(4,5)P_2_, whereas PI(3,4,5)P_3_ raised it by 80% (Group 3).^21^ Thus, each of the thirteen Kir channels could be grouped into one of four subtypes depending on the relative sensitivity to the three PIPs, while they still comprise one large group in that they are all sensitive to PI(4,5)P_2_.^21^ These specific PIP sensitivities indicate that the PIP binding and activation mechanisms are possibly controlled by far more subtle interactions besides the long-range electrostatics, and hence inspire more elaborate investigation on the ligand specificity.

In this study, we apply a rigorous free energy perturbation (FEP) method to model the three PIPs (*i.e.*, PI(4,5)P_2_, PI(3,4)P_2_ and PI(3,4,5)P_3_) bound upon the chicken Kir2.2 channel to explore the underlying molecular mechanism of these PIPs' specificity. By gradually mutating the native agonist PI(4,5)P_2_ to PI(3,4,5)P_3_ or PI(3,4)P_2_, we measured the free energy changes of each mutant relative to the native ligand by thermodynamic cycles in both bound and free states. The FEP calculation shows a 13.9 kcalmol^–1^ (ΔΔ*G*) and 39.7 kcalmol^–1^ for PI(3,4,5)P_3_ and PI(3,4)P_2_, respectively, indicating that the variants are less specific to the Kir2.2 channel and confirming the experimental findings on the mouse Kir2.2.^21^ Our results also reveal that the mutation shifted the binding modes from what the native ligand possessed, which was accompanied by the unfavorable solvation environment change around the ligands which eventually affected the channel gating efficiency.

## Methods

### Molecular model construction

The receptor–ligand complex of Kir2.2 and PI(4,5)P_2_ was initially configured based on the X-ray crystal structure of a Kir2.2 complexed with a short-chain dioctanoyl derivative of PIP_2_ (phosphatidylinositol biphosphate; PDB code: ; 3SPI).^18^ The missing aliphatic chains of PI(4,5)P_2_ molecules as well as missing atoms of the protein were constructed by using the Discovery Studio (Accelrys Inc., San Diego, CA, USA) software. The complete receptor–ligand model was subjected to energy minimization using the CHARMM program with an implicit membrane/solvent Generalized Born (GB) model for 1000 steps of steepest descent minimization in order to avoid steric clashes. The receptor–ligand complex was then inserted into a lipid bilayer of 437 1-palmitoyl-2-oleoyl-*sn-glycero*-3-phosphocholines (POPCs) by using the CHARMM-GUI membrane builder.^22^ The POPC membrane was widely used in the MD simulations for studying K channels.^23^–^26^ The final system was prepared after charge neutralization and immersion in 150 mM KCl solution of >57 000 TIP3P water molecules,^27^ resulting in a system of >250 000 atoms in a box of 140×140×160 Å^3^ with the membrane normally aligned on the *z*-axis.

### Molecular dynamics simulations

Inter- and intramolecular potential energies were calculated based on CHARMM36 force field.^28^,^29^ The van der Waals interactions were treated with a typical cutoff distance of 12 Å, while the long-range electrostatic interactions were estimated with the particle-mesh Ewald method.^30^ Molecular dynamics (MD) simulations were carried out with the NAMD2 software^31^ massively parallelized for IBM Blue Gene^32^,^33^ with a 2fs time step in a semi-isotropic isobaric and isothermal (NPT) ensemble of 1 atm and 303.15 K, by which the lateral and perpendicular dimensions of the membrane fluctuate independently. The system was first energy minimized for 10 000 steps with several restraints on the POPC membrane in order to retain the *cys* conformation of the unsaturated bond in the oleoyl chain, the stereochemical configuration (*i.e.*, R-form) of the glycerol linker as well as the membrane planarity. The system was further pre-equilibrated for 0.5 ns with a 1 fs time step, as the restraints imposed on the POPC membrane were gradually removed in multiple steps before the production runs. Then, we ran regular molecular dynamics simulations with no further restraints for over 80 ns with a 2 fs time step.

### Free energy perturbation calculation

The binding affinity changes were calculated by using the alchemical free energy perturbation method. Briefly, the free energy for a given state of a system can be defined with the Helmholtz formula,1
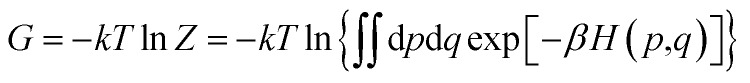
where *Z* and *H*(*p*,*q*) represent the partition function and the Hamiltonian of the system, respectively. The total free energy between two states of interest can be enumerated by summing the fractional free energy changes of multiple small steps from the initial state all the way to the final one through the alchemical mutation,2
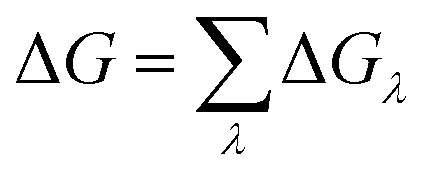

3 Δ*G*_*λ*_ = –*kT* ln ln〈exp(−exp(–*β*[*V*(*λ*+Δ*λ*)–*V*(*λ*)]))])〉*λ*

Where *V*(*λ*)=(1–*λ*)*V*_1_+*V*_2_, and *V*_1_ and *V*_2_ indicate the potential energies of two end states of our interest. In the alchemical mutation, the parameter *λ* is varied from 0 (state 1) to 1 (state 2) along multiple small steps. In our simulations, we employed 21 windows with soft-core potentials (*i.e.*, *λ* = 0, 0.00001, 0.0001, 0.001, 0.01, 0.05, 0.1, 0.2, 0.3, 0.4, 0.5, 0.6, 0.7, 0.8, 0.9, 0.95, 0.99, 0.999, 0.9999, 0.99999, 0.999999). The = 0, 0.00001, 0.0001, 0.001, 0.01, 0.05, 0.1, 0.2, 0.3, 0.4, 0.5, 0.6, 0.7, 0.8, 0.9, 0.95, 0.99, 0.999, 0.9999, 0.99999, 0.999999). The 〈⋯〉··· = 0, 0.00001, 0.0001, 0.001, 0.01, 0.05, 0.1, 0.2, 0.3, 0.4, 0.5, 0.6, 0.7, 0.8, 0.9, 0.95, 0.99, 0.999, 0.9999, 0.99999, 0.999999). The 〈⋯〉_*λ*_ represents the ensemble average of the potential energy difference over all available conformations at a given window *λ*. In each FEP*λ*-window, we obtained 200 000 conformations for the ensemble average with a 2 fs time step after the first 4000 steps of equilibration. The current setup provided a reasonable convergence for the binding affinity from our previous tests with larger window sizes and longer simulations.^34^–^37^ In order to avoid the singularity at the ends (so-called “end-point catastrophe”), the van der Waals potential for perturbed atoms was replaced with a soft-core modified 12-6 Lennard-Jones function,^36^,^37^ and the electrostatic interactions were switched on (or off) for the appearing (or disappearing) atoms after *λ* > 0.1 (or *λ* < 0.9) so as for the soft-core potential to repel possible atom overlapping.

In our study, the binding affinity for each non-native ligand (*i.e.*, PI(3,4,5)P_3_ or PI(3,4)P_2_) was calculated relative to the native agonist PI(4,5)P_2_*via* the thermodynamic cycle between the bound (*i.e.*, Kir2.2 and ligand) and free states (*i.e.*, ligand only) (Fig. 1b). The initial structure for the bound state was obtained from one of the last snapshots of the MD trajectory with Kir2.2 bound with PI(4,5)P_2_ in the POPC membrane. For the free state, we utilized one last snapshot with only PI(4,5)P_2_ in the POPC membrane after 20 ns MD simulation in the same electrolyte solution (*i.e.*, 150 mM KCl) as the bound state. For each state, we performed at least 5 independent runs from different initial configurations for better convergence. Thus, at least an 84 ns (21 windows × 0.4 ns × 5 runs × 2 states) long trajectory was generated for each ligand.

## Results and discussion

**Fig. 2:**
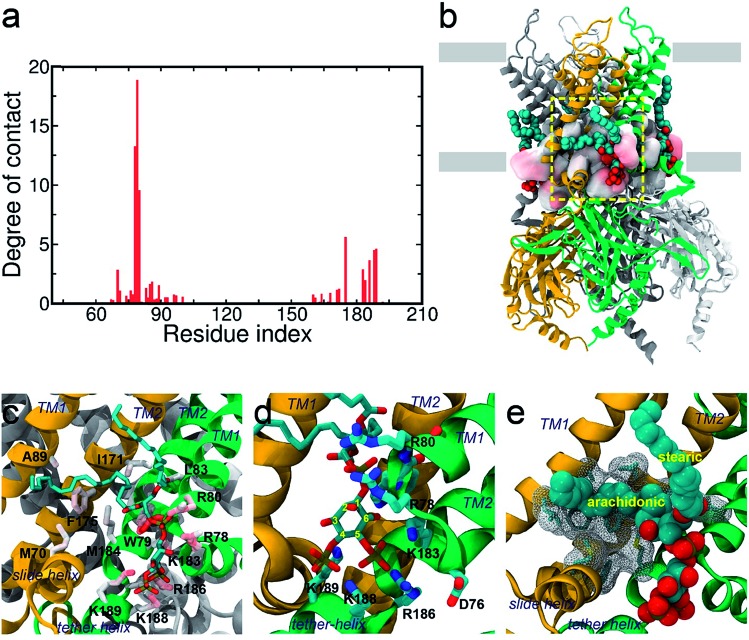
Binding mode of PI(4,5)P_2_ on KIR2.2. (a) Residue-specific atomic contact ratio of PI(4,5)P_2_ bound on KIR2.2. (b) A structural snapshot at about 85 ns. PI(4,5)P_2_ is stably bound at the putative binding sites located on the cytosolic surface of the POPC membrane. (c–e) A detailed binding mode for PI(4,5)P_2_ on KIR2.2. The negatively charged head group of PI(4,5)P_2_ is stabilized by a multitude of favorable salt-bridge interactions (c and d). The arachidonic chain seems to have favorable interaction with hydrophobic residues, while the stearic chain prefers to interact with lipid molecules (d).

In order to investigate the intrinsic binding mode between Kir2.2 and PI(4,5)P_2_, we first performed all-atom MD simulation on the Kir2.2–PI(4,5)P_2_ complex for over 80 ns (Fig. 1a). The channel complex as well as each subunit remains stable through the entire simulation, as indicated in the stable root mean square deviation (RMSD) fluctuation and consistent root mean square fluctuation (RMSF) patterns (Fig. S1†).The overall binding mode is summarized in Fig. 2a and b. The native agonist, PI(4,5)P_2_, maintains its stable position bound to the putative binding site near the interface between the cytoplasmic and transmembrane domains, and between the two adjacent channel subunits. Residue-specific atomic contacts indicate that the residues actively involved in the binding of PI(4,5)P_2_ include regions like the Slide helix (R65-R78), TM1(W79-A105), TM2 (A158-R186) and tether-helix (P187-T193). In particular, several high contacts appear in the middle of the Slide helix to the N-terminus of TM1, and the KKR motif of tether-helix. Site-directed mutagenesis studies confirmed the importance of these residues, indicating that their mutations either kill the channel activity or diminish the channel current drastically.^19^

### Binding mode of the native agonist PI(4,5)P_2_ on Kir2.2

#### Binding mode of the inositol head group

The putative binding site of Kir2.2 is characterized by a group of highly conserved positive residues such as R78, R80, K183, R186, K188 and K189. During our simulation, the inositol head group of each PI(4,5)P_2_ retained its crystal conformation at the putative binding site (see Fig. 2d). In more detail, the 5′- and 4′-phosphate groups are both involved in the binding site along with the residues K183, R186, K188 and K189 by favorable salt-bridges. This makes the inositol ring parallel to the plane generated by the inner and outer helices, efficiently mediating the helices. Also, this stabilizes the otherwise unstructured N-terminus (*i.e.*, K188 and K189) of the tether helix, tightly holding the CTD toward the TMD, and thus facilitating the open conformation.

#### Binding mode of the phosphodiester linker

In addition to the specific binding involving the inositol head group, our simulation also reveals the importance of the phosphodiester 1′-phosphate linker of PI(4,5)P_2_ and its contribution to the ligand binding stability. Even though the phosphodiester 1′-phosphate is located distantly from the inositol binding site, it is still capable of making a strong interaction with the TMD residues R78, W79, and R80 through long-range electrostatic interactions. In fact, these residues are the top three contributors to the binding stability out of all residues in contact with PI(4,5)P_2_ (see Fig. 2a). The persistent contacts are largely due to the concerted electrostatic interactions, with two salt-bridges (with R78 and R80) and multiple backbone hydrogen bonds (with R78 and W79) oriented toward the phosphodiester 1′-phosphate linker. Our observations are confirmed by the X-ray co-crystallized structure with dioctanoyl glycerol pyrophosphatide acid (PPA), where PPA can bind to this site even without the inositol head group.^18^ This implies that the phosphodiester 1′-phosphate plays a critical role in anchoring PI(4,5)P_2_. Therefore, in addition to introducing the inositol head group to the putative binding site, the phosphodiester 1′-phosphate also helps stabilize the binding. Our result also provides an important rationale on the highly conserved residues (*i.e.*, Arg(Lys)-Trp-Arg) among the eukaryotic Kirs, although they are not directly involved in channel gating.^18^

#### Binding mode of acyl chains

Besides the directional interaction through the charged phosphates, it is particularly noteworthy that the lipid acyl chains also appear to interact with Kir2.2 in a specific manner. Intriguingly, the contact analyses reveal that the acyl chains, even with the hydrophobic nature and intrinsic flexibility, seem to have specific contacts with the TMD residues. Such a tendency was more pronounced in the arachidonic chain rather than the stearic one, possibly owing to a different degree of bond saturation. For instance, the fully saturated stearic chain tends to dissolve in the POPC lipid bilayer rather than in contact with the channel. Even when it is in direct contact with the protein, it appears to prefer random contacts. On the other hand, the arachidonic chain with four double bonds tends to reside longer near Kir2.2, occupying the hydrophobic groove spanned over the surface composed of the inner and outer helices as well as the interfacial helix from the neighboring subunit (Fig. 2e). Although not as specific as the inositol head or the phosphodiester linker, several hydrophobic hot spots were recognized for the arachidonic chain: M70, L83, L85, F86, A89, I171, I172, F175 and M184. In particular, F175 was found as the highest contacting residue next to the three pivotal residues (*i.e.*, R78, W79 and R80) for the phosphodiester linker.

The saturation state of acyl chains was proposed as an important factor for the channel signaling mechanism. The replacement of the unsaturated arachidonic chain with a saturated one changed the partition of the signaling lipids (*i.e.*, ligand) between the channel protein and lipid raft.^38^ More directly, the chemical diversity of acyl lipids may affect the binding affinity and gating, as shown in the hydrophobic interaction of PI(4,5)P_2_ activation of Ca_v_2.2.^39^ Our results provide an important insight into the role of acyl chains in stabilizing (or supporting) the ligand binding which can subsequently influence channel gating.

### Phosphoinositide specificity for Kir2.2 by free energy perturbation

Next we turned our focus onto the ligand specificity for Kir2.2 between the native ligand PI(4,5)P_2_ and the two common variants PI(3,4)P_2_ and PI(3,4,5)P_3_. Using FEP, we assessed the binding affinity changes for the mutations from PI(4,5)P_2_ to PI(3,4,5)P_3_ or to PI(3,4)P_2_. Based on the thermodynamic cycle shown in Fig. 1b, the relative binding free energy changes, ΔΔ*G* from PI(4,5)P_2_ to PI(3,4,5)P_3_/PI(3,4)P_2_ (ΔΔ*G*_WT→MUT_), were obtained by performing multiple FEP simulations for both the bound state (Δ*G*_bound_) and free state (Δ*G*_free_). Table 1 summarizes the FEP calculation results. For the mutation from PI(4,5)P_2_ to PI(3,4,5)P_3_, ΔΔ*G* = 13.9 kcalmol^–1^, whereas for the mutation from PI(4,5)P_2_ to PI(3,4)P_2_, ΔΔ*G* = 39.7 kcalmol^–1^, indicating a strong preference of PI(4,5)P_2_ over PI(3,4,5)P_3_ or PI(3,4)P_2_ (least preferred). Our results are in good agreement with the experimental findings, where PI(3,4,5)P_3_ shows only ∼30% activation of Kir2.2 as compared to the wide-type PI(4,5)P_2_, while PI(3,4)P_2_ shows no activation.^21^

**Table 1:** FEP results for (a) PIP45 to PIP345 and (b) PIP45 to PIP34

Mutation	PI(4,5)P_2_ to PI(3,4,5)P_3_				PI(4,5)P_2_ to PI(3,4)P_2_			
	Δ*G*	Δ*G*_elec_^*a*^	Δ*G*_vdW_^*b*^	Δ*G*_couple_^*c*^	Δ*G*	Δ*G*_elec_^*a*^	Δ*G*_vdW_^*b*^	Δ*G*_couple_^*c*^
Bound	–277.6 (1.2)^*d*^ ^,^^*e*^	–310.6 (1.5)	25.1 (0.4)	7.9 (0.2)	64.3 (2.6)	60.4 (2.5)	–12.7 (0.6)	16.6 (0.6)
Free	–291.5 (0.8)	–324.3 (0.9)	23.1 (0.6)	9.7 (0.1)	24.6 (1.7)	20.3 (1.8)	–12.6 (0.4)	17.0 (0.4)

	ΔΔ*G*^*d*^ ^,^^*e*^	ΔΔ*G*_elec_	ΔΔ*G*_vdW_	ΔΔ*G*_couple_	ΔΔ*G*	ΔΔ*G*_elec_	ΔΔ*G*_vdW_	ΔΔ*G*_couple_
Total	13.9 (1.4)	13.7 (1.8)	2.0 (0.7)	–1.8 (0.3)	39.7 (3.1)	40.1 (3.1)	0.0 (0.7)	–0.3 (0.8)

^*a*^Electrostatic contribution of free energy.

^*b*^van der Waals contribution of free energy.

^*c*^Coupling terms are obtained from Δ*G*_couple_ = Δ*G*– Δ*G*_elec_– Δ*G*_vdW_.

^*d*^ΔΔ*G* = Δ*G*_bound_– Δ*G*_free_.

^*e*^Standard errors.

In the normal physiological environment, the inositol head group of PIP_2_ and PIP_3_ can hold four and six negative charges, respectively. This implies that the electrostatic interaction might play a critical role in PI(4,5)P_2_ binding to Kir2.2. To further explore this, we compared the energetic components of the total binding free energies (Table 1). Expectedly, both mutations showed that the electrostatic component dominates the free energy changes. For example, in the mutation to PI(3,4,5)P_3_, the total electrostatic and van der Waals contributions to the binding free energy of ΔΔ*G* = 13.9 kcalmol^–1^ are 13.7 and 2.0 kcalmol^–1^, respectively, with –1.8 kcalmol^–1^ for the coupling term. Here, we found that the mutation stabilizes the ligand electrostatically, particularly, in the free state, which implies that the strong binding specificity of PI(4,5)P_2_ over PI(3,4,5)P_3_ originates indirectly from the partitioning difference between the bound and the free states, rather than from destabilization of the ligand in the bound state.

For the mutation from PI(4,5)P_2_ to PI(3,4)P_2_, similar to that to PI(3,4,5)P_3_, it also shows that the total free energy change of ΔΔ*G* = 39.7 kcalmol^–1^ is largely due to the electrostatic free energy change, which is ΔΔ*G*_elec_ = 40.1 kcalmol^–1^. However, the rationality for the ligand specificity in this case is quite different from the mutation to PI(3,4,5)P_3_. Here, the specific binding for PI(4,5)P_2_ is directly related to the unfavorable ligand stability in the bound state, as compared to PI(3,4)P_2_. In other words, the translocation of one phosphate from 4′ to 3′ position hugely destabilized the binding. Given the equivalent charges between the two ligands, our finding strongly indicates that the binding specificity of PI(4,5)P_2_ is regulated by a more sophisticated local binding mode (see below for the details).

### Binding mode shift by PI(4,5)P_2_ to PI(3,4,5)P_3_ mutation

We next explored the underlying mechanism of the unfavorable free energy changes upon mutations of PI(4,5)P_2_ into the non-native PI(3,4)P_2_ and PI(3,4,5)P_3_. As described in the aforementioned sections, X-ray crystallography data and MD simulations have indicated that PI(4,5)P_2_ interacts with Kir2.2 at the binding site in a sophisticated and well-coordinated way. During the interaction, both 4′- and 5′-phosphates simultaneously form salt bridges with K188 and K189 (KKR motif on the tether helix). Towards the center of the channel, 5′-phosphate also efficiently interacts with K183 (in the TM2) and R186. On the other hand, 1′-phosphate, located at the membrane interface, remains anchored by R78, W79 and R80 (the three residues linking the Slide helix and TM1). These residues were reported to be critical in other Kir channels (their mutations would either kill the channel activities or dramatically reduce the ligand sensitivities^19^).

**Fig. 3:**
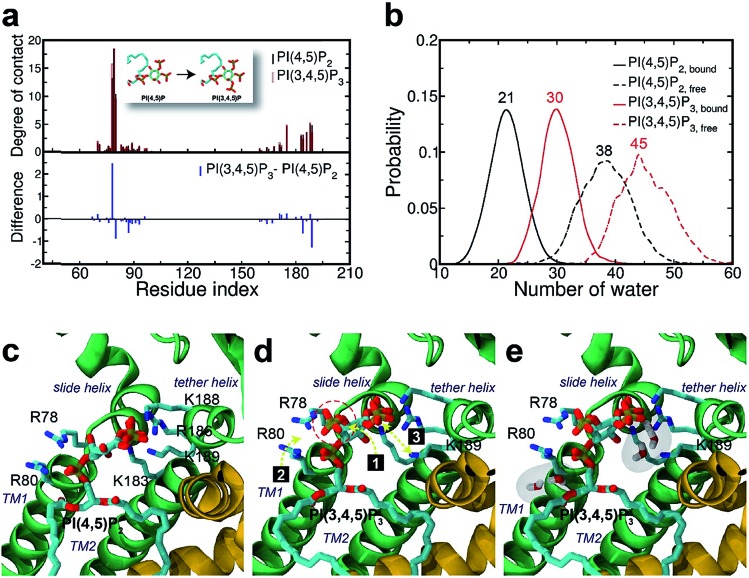
Binding mode change by mutation from PI(4,5)P_2_ to PI(3,4,5)P_3_. (a) Residue-specific atomic contact ratio (up) and changes (down) upon the phosphoinositide ligand mutation. (b) Water distribution changes around phosphoinositide ligands. Numbers on each graph indicate average number of water molecules in the first solvation shell. (c) Binding mode of PI(4,5)P_2_. (d) Binding mode after mutation to PI(3,4,5)P_3_. Number bullets (1 to 3) describe a schematic order for the binding mode shift upon the mutation. (e) Binding mode shift with PI(3,4,5)P_3_ leaves room for water intrusion near K189 and R80.

As PI(4,5)P_2_ mutates into PI(3,4,5)P_3_, the binding pose gets slightly shifted from the native binding configuration albeit without much difference (Fig. 3c and d). For example, superimposition of a representative chain shows that the inositol phosphates P1, P4 and P5 of PI(3,4,5)P_3_ have been displaced by only 0.9, 0.8 and 1.5 Å, respectively, from those of PI(4,5)P_2_. Also, a majority of the salt bridges appeared to be well preserved (*e.g.*, K183-P5, R186-P5, and K188-P4). Yet, residue-specific atomic contacts reveal that there are several significant differences between the PI(4,5)P_2_ and PI(3,4,5)P_3_ complexes (Fig. 3a and Table S1†). We highlighted the residues showing remarkable contact change by the residue contact probability difference (Δ*q* = *q*_MUT_–*q*_WT_). The residue-specific atomic contact is defined as the sum of each atomic contact probability of a chosen residue, *i.e.*, 
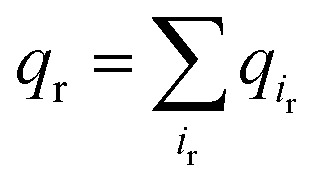
, where the atomic contact probability,
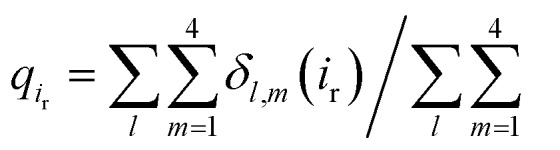
, of a heavy atom *i*_r_ of a residue r was obtained by counting the atomic contact *δ*_*l*,*m*_(*i*_r_) with a 5 Å cut-off from any ligand heavy atoms, over all 4 channel subunits and all MD frames (counted by *m* and *l* respectively). Taking |Δ*q*| > 0.5 as a criterion, K189, R80, M184 and S87 substantially decreased in the ligand contact by –1.25, –0.85, –0.64 and –0.59, respectively. Meanwhile, there is a huge increase (Δ*q* = 2.50) for R78, which is one of the anchoring residues to the phosphodiester linker. In fact, even with one more phosphate, the overall atomic contacts (Δ*q*_all_ = Δ*q*_(–)_ + Δ*q*_(+)_ = –5.65 + 4.59 = –1.06) between PI(3,4,5)P_3_ and Kir2.2, compared with PI(4,5)P_2_, are decreased, implying a weakened connection between PI(3,4,5)P_3_ and Kir2.2. This observation is also reflected by the site-directed mutagenesis experiments, which show that neutralizing one of these basic residues decreases the EC_50_ of PI(4,5)P_2_ to Kir2 channels.^19^

As can be seen in Fig. 3d, the additional phosphate at 3′ position can increase the electrostatic attraction to R78. The interaction is so effective that it accounts for as much as 50% of the total contact increase. Nonetheless, our simulation showed that the strong attraction between R78 and 3′-phosphate group rather destabilized the ligand binding in at least two different ways. First, it slightly distorted the inositol head group from its native binding conformation. This in turn substantially weakened the salt-bridge between 5′-phosphate and K189, as evidenced in the significant reduction of K189 contact (*i.e.*, Δ*q* = –1.25) as well as elongation of the salt-bridge distance. For example, the distance between K189 and 5′-phosphate in one representative structure was found to be elongated from 2.5 Å to 5.7 Å (only heavy atoms counted) after the mutation. Second, it also disturbed the binding of the phosphodiester linker. As 3′-phosphate begins to interact with R78, the interaction between 1′-phosphate and R80 becomes more unstable, as indicated in the decreased contact (*i.e.*, Δ*q* = –0.85).

To further investigate the potential impact of key residues on PIPs' binding, we selected two PI(4,5)P_2_ binding residues, K189 and R80, to mutate to alanine, respectively, and conducted the corresponding FEP calculations as the wildtype (Table S3†). For the K189A mutant, the free energy changes for PI(4,5)P_2_ to PI(3,4,5)P_3_, ΔΔ*G*_PIP2→PIP3_ is 15.0 kcalmol^–1^; and for the R80A mutant, the ΔΔ*G*_PIP2→PIP3_ is 12.9 kcalmol^–1^. Comparing the wildtype's ΔΔ*G*_PIP2→PIP3_ of 13.9 kcalmol^–1^, the Lys-deletion from the residue 189 favors PI(4,5)P_2_ relatively more than PI(3,4,5)P_3_ by about 1 kcalmol^–1^; while the Arg-deletion from the residue 80 reduces the binding affinity of the native ligand PI(4,5)P_2_ by about 1 kcalmol^–1^. Although two mutations are, obviously, not sufficient to draw a conclusion, the binding site residues may play a different role in the ligand binding depending on their location and binding contribution. For example, K189 may contribute more to the binding selectivity since it is located near the phosphoinositide head group, while R80 may contribute more to the binding stability due to its role in coordinating to the phosphodiester linker.

Furthermore, we also found that the water environment can be altered with PI(3,4,5)P_3_ from that with PI(4,5)P_2_. In the free state, on average 38 water molecules were found in the first hydration shell of PI(4,5)P_2_, whereas 7 more were found in that of PI(3,4,5)P_3_ (*i.e.*, 45 water molecules), possibly due to the additional phosphate group. However, in the bound state, we found 9 more water molecules (*i.e.*, 30 water molecules) around PI(3,4,5)P_3_ compared to that of PI(4,5)P_2_ (*i.e.*, 21 water molecules). In other words, on average 17 water molecules (38–21) have been desolvated from PI(4,5)P_2_ upon binding to Kir2.2, whereas only 15 water molecules (45 – 30) have been desolvated from PI(3,4,5)P_3_. This may indicate that PI(3,4,5)P_3_ is in a relatively loose coupling with Kir2.2 despite the increased charge (*i.e.*, –6e in the inositol head). In fact, we were able to identify two regions where water can intrude for the case of PI(3,4,5)P_3_. One region is found near the inositol head, *i.e.*, a space formed after the salt-bridge breaks between K189 and 5′-phosphate (Fig. 3e). The other was near the phosphodiester linker, *i.e.*, a gap between the lipid bilayer and R80. In addition to water molecules, divalent ions in the local environment may also influence the stability of PIPs by forming stable interactions with phosphate groups,^40^ however, the typical concentration of divalent ions (*e.g.* 1 mM MgCl_2_ in electrophysiological experiments) is usually too low to have a meaningful effect on PIPs' binding to Kir channels. In summary, the 3′-phosphate of PI(3,4,5)P_3_ introduces a new interaction between R78 and the inositol head group. However, it can disturb the native binding mode of the inositol head to Kir2.2, especially the salt-bridge between 5′-phosphate and K189. Also, it can perturb the interaction between 1′-phosphate and R80 (Fig. 3d), which effectively weakens the ligand binding accompanied by the loose desolvation, and eventually the gating function for Kir2.2.

### Binding mode shift by PI(4,5)P_2_ to PI(3,4)P_2_ mutation

Surprisingly, the mutation to PI(3,4)P_2_ impacted more dramatically than PI(3,4,5)P_3_, on the native binding mode, resulting in a more apparent shift from the native one. For example, the displacements for equivalent phosphates (*i.e.*, P1 and P4) of PI(3,4)P_2_ from PI(4,5)P_2_ were 2.1 and 1.8 Å, respectively, each increased at least 1.0 Å when compared to the corresponding pairs between PI(4,5)P_2_ and PI(3,4,5)P_3_. Although PI(3,4)P_2_, as a configurational isomer of PI(4,5)P_2_, has only one phosphate switched from 5′ to 3′ of the inositol ring, the 3′-phosphate was not having strong enough interaction to substitute for that mediated by the 5′-phosphate as in PI(4,5)P_2_. More specifically, the distances of K183, R186, and K188 to 5′-phosphate of PI(4,5)P_2_ were 2.7, 2.6, and 2.6 Å, respectively, whereas those to 3′-phosphate of PI(3,4)P_2_ have been elongated to 5.6, 7.2, and 4.8 Å, respectively. On the other hand, the 3′-phosphate can form a new salt bridge with R78 with a distance of 2.6 Å. However, this new single salt-bridge seriously disturbs the stable salt-bridge network, especially the ones formed between 1′-phosphate and R78/R80, and between 4′-phosphate and K188 (*e.g.*, a distance change from 5.6 to 8.4 Å), thus resulting in a net loss in terms of interaction with the protein.

This change is also reflected in a substantial reduction of contact probabilities (*i.e.*, Δ*q* < –0.5) for a majority of binding site residues, including R80, K183, R186, K188 and K189. Of course, R78 has increased its contact somewhat by Δ*q* = 1.19, similar to the case of PI(3,4,5)P_3_, largely due to the new salt-bridge from the 3′-phosphate of PI(3,4)P_2_ mentioned above. Nevertheless, as expected, the overall residue-contact has been greatly reduced by Δ*q*_all_ = –6.05 (=Δ*q*_(+)_ + Δ*q*_(–)_ = 3.26 + (–9.31)) from the mutation to PI(3,4)P_2_. These findings, together with the above FEP results, indicate serious destabilization in both PI(3,4,5)P_3_ and PI(3,4)P_2_, but with a significantly more deleterious impact from PI(3,4)P_2_.

**Fig. 4:**
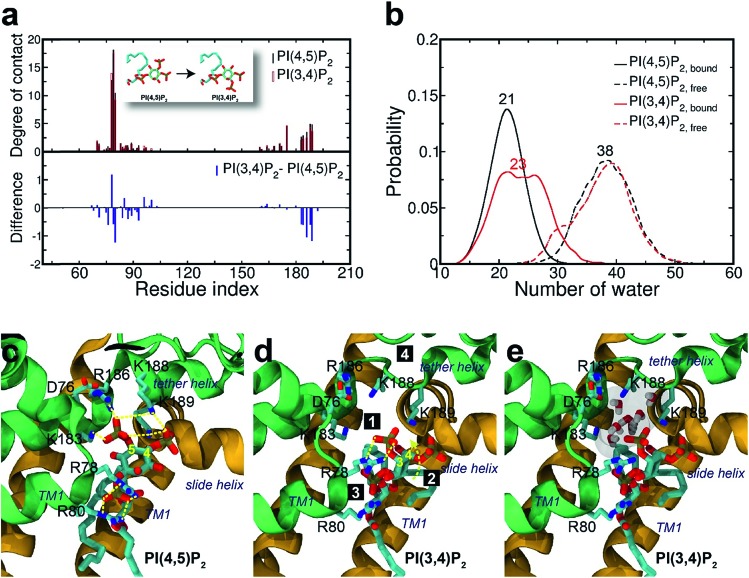
Binding mode change by mutation from PI(4,5)P_2_ to PI(3,4)P_2_. (a) Residue-specific atomic contact ratio (up) and changes (down) upon the phosphoinositide ligand mutation. (b) Water distribution changes around phosphoinositide ligands. Numbers on each graph indicate the average number of water molecules in the first solvation shell. (c) Binding mode of PI(4,5)P_2_. (d) Binding mode after mutation to PI(3,4)P_2_. Number bullets (1 to 4) describe a schematic order for the binding mode shift upon the mutation. (e) Binding mode shift with PI(3,4)P_2_ leaves a large room for water intrusion between the ligand and the positive residues (*i.e.*, K183, R186, and K188) in the binding site. Note that the tether helix has been relaxed with PI(3,4)P_2_.

The larger perturbation was also evidenced by the water distribution changes in the first solvation shell of PI(3,4)P_2_. In the free state, PI(3,4)P_2_, as a configurational isomer of PI(4,5)P_2_, displays more or less similar water distribution with an average of 38 water molecules around the ligand (Fig. 4b). In the bound state, however, more water molecules (also with wider fluctuation) surround PI(3,4)P_2_, resulting in a stronger solvation than that observed in the native ligand PI(4,5)P_2_. We found that the increased solvation of PI(3,4)P_2_ in the bound state was mainly from the deletion of 5′-phosphate. As aforementioned, the deletion removed the important interaction between the phosphate and tether helix, leaving a large space behind. The newly substituted 3-phosphate group tried to compensate for the loss by tilting its binding slightly toward the helix, but it was not sufficient to cause any significant impact. Instead, water molecules entered this space and stabilized the exposed charged residues (*i.e.*, K183, R186 and K188) of the tether helix, as shown in Fig. 4e, where seven water molecules filled in the open space, interacting with the nearby positive residues.

### Structural impact on the tether helix

Furthermore, we noticed that the tether helix loses its helical structure immediately after it loses the salt-bridge interactions with the ligand upon the 5′-phosphate deletion. Comparing Fig. 4c and d, the first helical turn is relaxed to a random coil after the PI(4,5)P_2_ to PI(3,4)P_2_ mutation. Fig. 4d summarizes the binding mode shift along the mutation from PI(4,5)P_2_ to PI(3,4)P_2_: (1) P3 group shows up with 5′-phosphate deleted; (2) the inositide group of PI(3,4)P_2_ rotates slightly in order to fit the binding site, leaving a large room for water intrusion; (3) new salt bridges formed between R78 and 3′-phosphate; and (4) interactions between PI(3,4)P_2_ and K183/R186/K188 are weakened, accompanied by the relaxation of the tether helix to a random coil.

**Fig. 5:**
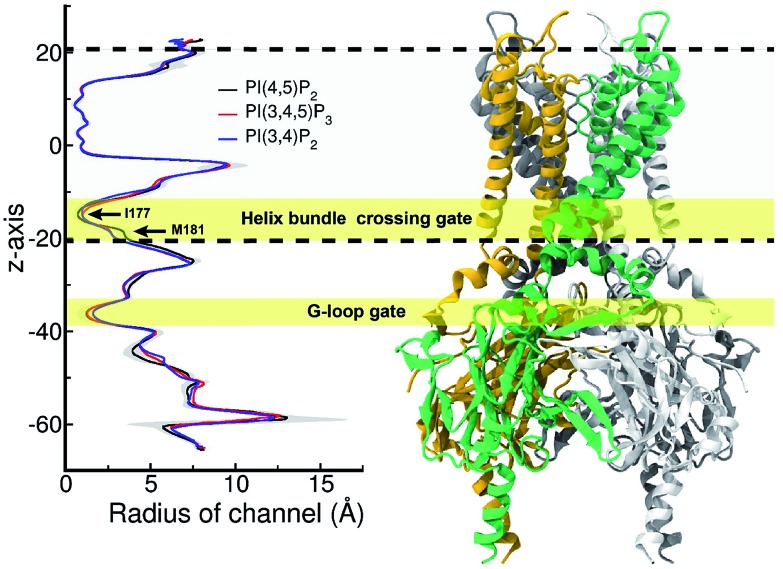
Pore radius change upon change in the type of phosphoinositide. ore-radii are sensitive to the types of phosphoinositides especially at the helix bundle crossing (HBC) and G-loop gates. In particular, for the HBC gate, the channel near M181 gets narrowed by PI(3,4,5)P_3_ and PI(3,4)P_2_, while it is more opened by the wild-type PI(4,5)P_2_. The region between two dashed lines indicates TMD of KIR2.2.

Fig. 5 shows the pore radius change upon the mutations to both PI(3,4,5)P_3_ and PI(3,4)P_2_. As shown in the figure, pore-radii are sensitive to the types of phosphoinositides at the helix bundle crossing (HBC) and G-loop gates. In particular, for the HBC gate, the channel near M181 gets narrowed by PI(3,4,5)P_3_ and PI(3,4)P_2_, while it is more opened by the wild-type PI(4,5)P_2_. Thus, even though no large overall conformational changes were observed in the channel (see Fig. 5), the local interactions especially by the 5′-phosphate group of PI(4,5)P_2_ with key residues K183/R186/K188 did play a critical role in maintaining the channel pore size. It is worth noting that K183 is a highly conserved residue among the Kir family. In Kir2.1, even mutation to arginine might cause channel malfunction, and mutation to glutamine seriously reduces the sensitivity of PI(4,5)P_2_.^19^ Our previous MD simulations on Kir3.1 chimera also showed that K183 tends to form more stable salt bridges with PI(4,5)P_2_ in the G-loop open state than in the closed one, suggesting a close relationship between K183 and PI(4,5)P_2_ for the channel opening.^15^ Consistently, absence of interaction with K183 in PI(3,4)P_2_ may constitute a reason for its disability to activate the channel. R186 was also experimentally examined in Kir2.1 that affects PIPs' specificity: R186Q mutation is able to recover ∼20% activity of Kir2.1 under PI(3,4,5)P_3_, but not under PI(3,4)P_2_.^21^ This can be explained by our current simulation. R186, located at the end of TM2, forms a very stable salt-bridge with 5′-phosphate of PI(4,5)P_2_ partly by a stable holding for R186 from behind (*i.e.*, *via* ε-N or one of the η-N's of the guanidinium group) with D76 which is also a conserved residue located in the C-terminus of the Slide helix. In the absence of 5′-phosphate of PI(3,4)P2, however, R186 turns toward D76 and forms a strong salt-bridge with D76 (2.9 Å between heavy atoms), using two η-N's. It was reported that the opening of channels involves a rotating motion of Slide helix.^17^,^18^,^41^,^42^ The salt bridge of R186-D76 thus might serve as a ‘lock’ for such rotation. Furthermore, glutamine instead of arginine at this position will lower the energy to unlock, and thus reducing the channel selectivity on PIPs (*e.g.* Kir6.2).

On the other hand, K188 is the first lysine in the highly conserved KKR motif of the following tether helix in the PIPs' binding region. Many studies have shown conformational changes of the motif (or tether-helix) from the loop to helix along with the opening of the Kir2 channels. PI(4,5)P_2_ induces this conformational change and promotes the channel opening.^18^,^42^ In our simulations we did observe the relaxation of the tether-helix, which is directly related to the loss of a series of important interactions (including with K188 and K189) in the PI(3,4)P_2_ complex, as discussed above.

### Effect on the channel contact of acyl chains

Lastly, in addition to the basic residues, some hydrophilic and hydrophobic residues that interact with the acyl chains also show a large decrease of their contact probabilities by mutation to PI(3,4,5)P_3_/PI(3,4)P_2_, including F71, L84, S87, A89, S93 and M184 (Table S1†). Most of these residues are mainly located in the TM1, except for F71 in the Slide helix and M184 in the TM2 (see Fig. 2). As discussed before, the arachidonic chain of PI(4,5)P_2_ can have a relatively stable interaction with the transmembrane helices. Our result indicates that phosphate substitution on the inositide can affect not only the binding mode of the inositol head group contributed mainly by strong and directed electrostatic interaction, but also by the hydrophobic tails through non-specific hydrophobic interaction. Our previous study on the Kir3.1 chimera also indicated that a hydrophobic core, composed of L68, F72 (corresponding to F71 in Kir2.2), V76, L175, F181 and M184 (corresponding to M184 in Kir2.2), helped stabilize the open state of the HBC gate.^43^ According to current results, the arachidonic chain of the native agonist PI(4,5)P_2_ also contributed to this hydrophobic core stabilization, while for PI(3,4)P_2_ and PI(3,4,5)P_3_, their changes on the head group alter their tails' orientations, which further diminishes their stabilization of the open HBC state.

## Conclusions

In this study, we carried out rigorous FEP simulations to investigate the binding events of three PIPs, PI(4,5)P_2_, PI(3,4,5)P_3_, and PI(3,4)P_2_, at the active site of Kir2.2. By gradually mutating PI(4,5)P_2_ to the other two PIPs, we demonstrated a weakened binding affinity to the Kir2.2 channel with ΔΔ*G* = +13.9 kcalmol^–1^ for PI(3,4,5)P_3_ and +39.7 kcalmol^–1^ for PI(3,4)P_2_ respectively, indicating a remarkable energetic advantage of PI(4,5)P_2_ in binding with Kir2.2 over the other two PIPs. These results are in good agreement with the previous experimental observations that the PI(4,5)P_2_ is the primary agonist to maintain the normal function of Kir2 channels while PI(3,4,5)P_3_ and PI(3,4)P_2_ hardly activate the channels.

Despite that these three PIPs share very similar chemical and structural properties, they show extremely different specificities in activating Kir2 channels with such dramatic binding affinity differences. Our further analyses revealed the underlying molecular mechanism for those differences. PI(4,5)P_2_ is capable of forming a series of critical salt bridges to Kir2.2 with each phosphate specifically corresponding precisely to one or two basic residues (*e.g.*, 1′-phosphate-R78/R80, 4′-phosphate-K188/K189, 5′-phosphate-R186/K188). However, either adding one more phosphate (PI(4,5)P_2_ to PI(3,4,5)P_3_) or changing the positions of phosphate (PI(4,5)P_2_ to PI(3,4)P_2_) cannot accurately reproduce these interactions, resulting in a substantial reduction of several critical interactions. Furthermore, the destabilization of PI(3,4)P_2_ was found to originate mainly from the unstable bound state, whereas PI(3,4,5)P_3_ binding is mostly affected by a more preferred partition for the free state. In addition to strong electrostatic interactions, we found that the arachidonic chain of PI(4,5)P_2_ also contributes to the lipid agonism *via* favorable hydrophobic core formation with nearby hydrophobic residues such as M70, L83, L85, F86, A89, I171, I172, F175 and M184.

Finally, it should be noted that previous studies have shown that PI(4,5)P_2_ controls the channel gates through allosteric regulation. It is known that the PI(4,5)P_2_ binding causes conformational changes of Kir2.2, such as a new helix formation in the N-terminal Slide helix from the disordered fragment, and another helix formation in the tether-helix from a loop, which is observed in our current simulation as well.^18^ However, a detailed molecular picture by which how PI(4,5)P_2_ binding can couple/regulate the Kir2 channel gating is still not available, which needs further studies in the future. Uncovering such a coupling mechanism would offer deeper insights to further interpret the specificity of PIPs to Kir channels. Finally, it should be mentioned that FEP can converge slowly particularly on systems where the environments of the target alchemical modification undergo slow response fluctuations; for this purpose, various advanced sampling strategies, such as Orthogonal Space Random Walk (OSRW), were developed^44^ to help reduce the enormous computational resources required and better understand the underlying mechanism of the PIP's specificity for potassium channels.

## Conflicts of interest

There are no conflicts to declare.

## Supplementary Material

Supplementary information
