# Supplementary material for: Molecular mechanism of phosphoinositides' specificity for the inwardly rectifying potassium channel Kir2.2
Source: Chem Sci. 2018 Sep 5;9(44):8352–62. doi: 10.1039/c8sc01284a (PMC6247517; doi:10.1039/c8sc01284a)
Supplement: Supplementary file 1 [file SC-009-C8SC01284A-s001.pdf]

Electronic Supplementary Material (ESI) for Chemical Science.
This journal is © The Royal Society of Chemistry 2018

Supporting Information For

Molecular Mechanism of Phosphoinositides' Specificity for the Inwardly Rectifying Potassium Channel Kir2.2

Xuan-Yu Meng[1,†], Seung-gu Kang[2,†], Ruhong Zhou[1,2,3,*]

[1]Institute of Quantitative Biology and Medicine, SRMP and RAD-X, Collaborative Innovation Center of Radiation Medicine of Jiangsu Higher Education Institutions, Soochow University, Suzhou 215123, China

[2]IBM Thomas J. Watson Research Center, Yorktown Heights, NY 10598, USA

[3]Department of Chemistry, Columbia University, New York, NY 10027, USA

†These authors equally contributed.

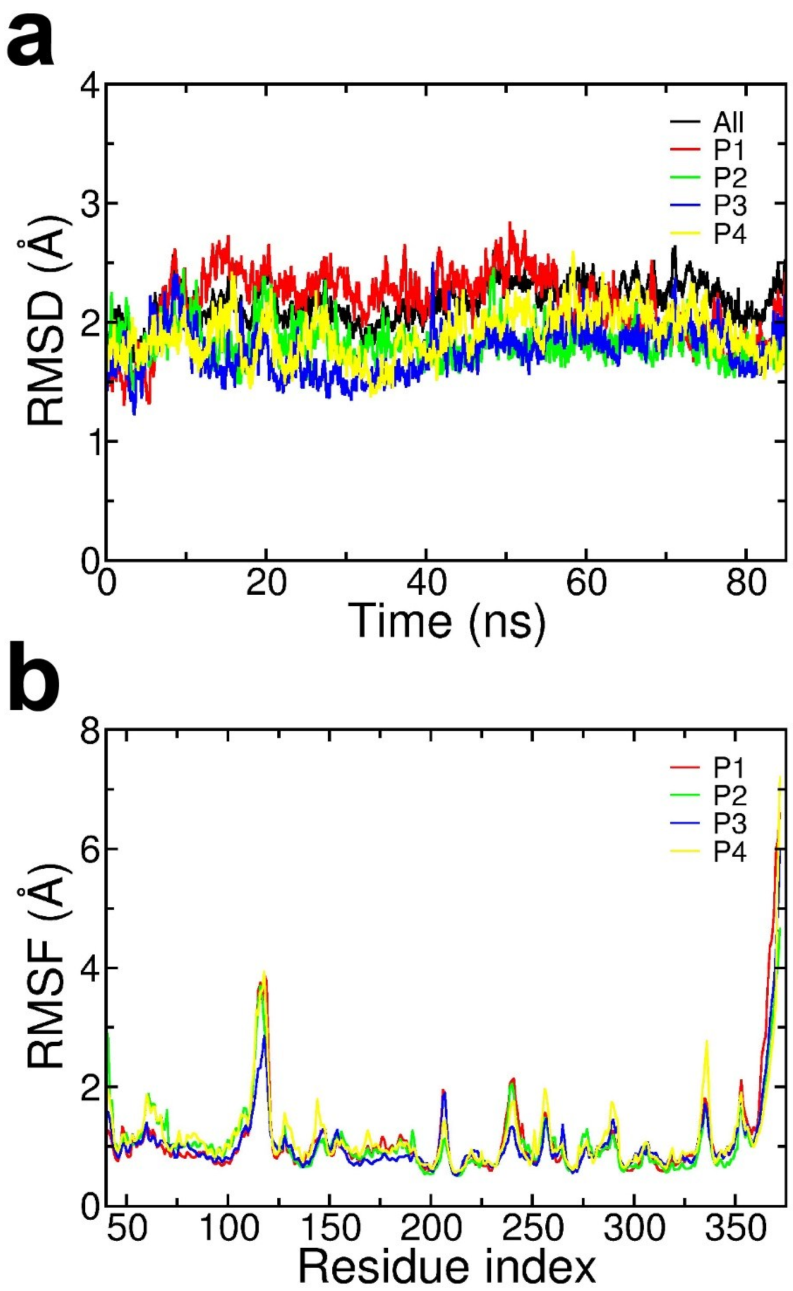

Figure S1 | Root-mean squared deviation and fluctuation for KIR2.2. a, RMSDs show that KIR2.2 bound with PI(4,5)P_2 is quite stable at each monomer or whole tetramer levels. **b**, RMSF displays stable residue fluctuation for all subunits except for the extracellular loop peaked at D117.

Table S1 | Residue contact change by mutation of PI(4,5)P_2 to PI(3,4,5)P_3

Residues of $\Delta q < 0$					Residues of $\Delta q > 0$				
Res	AA	q_{MUT}	q_{WT}	Δq	Res	AA	q_{MUT}	q_{WT}	Δq
68	ALA	0.090	0.142	-0.052	47	LYS	0.004	0.000	0.004
71	PHE	1.339	1.457	-0.118	49	ASN	0.031	0.000	0.031
74	CYS	0.253	0.286	-0.033	51	GLN	0.041	0.029	0.012
76	ASP	1.003	1.168	-0.165	67	ILE	0.127	0.009	0.118
80	ARG	9.575	10.426	-0.851	70	MET	1.956	1.715	0.241
83	LEU	1.303	1.401	-0.098	75	VAL	0.388	0.317	0.071
84	LEU	0.928	1.010	-0.082	77	ILE	0.745	0.685	0.060
86	PHE	0.934	1.062	-0.128	78	ARG	15.740	13.245	2.495
87	SER	0.561	1.155	-0.594	79	TRP	18.462	18.418	0.044
88	LEU	0.206	0.367	-0.161	81	TYR	0.063	0.046	0.017
89	ALA	1.496	1.698	-0.202	85	LEU	0.693	0.621	0.072
91	LEU	0.193	0.363	-0.170	90	PHE	0.002	0.001	0.001
92	VAL	0.564	0.617	-0.053	94	TRP	0.002	0.000	0.002
93	SER	0.930	1.158	-0.228	95	LEU	0.001	0.000	0.001
160	PHE	0.544	0.659	-0.115	96	LEU	0.667	0.537	0.130
161	MET	0.256	0.353	-0.097	97	PHE	0.659	0.629	0.030
163	VAL	0.065	0.109	-0.044	99	LEU	0.002	0.000	0.002
164	VAL	0.710	0.921	-0.211	100	ILE	0.194	0.192	0.002
167	ILE	0.101	0.274	-0.173	103	LEU	0.001	0.000	0.001
179	ALA	0.009	0.021	-0.012	168	VAL	1.020	1.011	0.009
184	MET	2.476	3.113	-0.637	171	ILE	1.657	1.395	0.262
186	ARG	3.515	3.599	-0.084	172	ILE	1.221	1.016	0.205
189	LYS	3.560	4.812	-1.252	174	SER	0.034	0.000	0.034
192	GLN	0.006	0.093	-0.087	175	PHE	4.816	4.559	0.257
					180	ILE	0.118	0.007	0.111
					183	LYS	3.070	2.930	0.140
					188	LYS	5.198	4.957	0.241

Table S2 | Residue contact change by mutation of PI(4,5)P_2 to PI(3,4)P_2

Residues of $\Delta q < 0$					Residues of $\Delta q > 0$				
Res	AA	q_{MUT}	q_{WT}	Δq	Res	AA	q_{MUT}	q_{WT}	Δq
51	GLN	0.027	0.057	-0.030	49	ASN	0.007	0.000	0.007
68	ALA	0.011	0.114	-0.103	64	GLN	0.001	0.000	0.001
71	PHE	1.095	1.436	-0.341	67	ILE	0.096	0.016	0.080
75	VAL	0.111	0.277	-0.166	70	MET	1.908	1.821	0.087
76	ASP	0.333	0.949	-0.616	73	THR	0.007	0.000	0.007
79	TRP	17.560	18.147	-0.587	74	CYS	0.337	0.313	0.024
80	ARG	9.226	10.466	-1.240	77	ILE	0.650	0.628	0.022
81	TYR	0.008	0.039	-0.031	78	ARG	13.947	12.762	1.185
84	LEU	0.505	0.871	-0.366	82	MET	0.003	0.000	0.003
86	PHE	1.090	1.164	-0.074	83	LEU	1.425	1.386	0.039
87	SER	0.678	1.083	-0.405	85	LEU	0.932	0.582	0.350
88	LEU	0.187	0.295	-0.108	90	PHE	0.006	0.001	0.005
89	ALA	1.337	1.624	-0.287	94	TRP	0.025	0.004	0.021
91	LEU	0.212	0.299	-0.087	96	LEU	0.973	0.586	0.387
92	VAL	0.459	0.620	-0.161	97	PHE	0.716	0.669	0.047
93	SER	0.673	1.125	-0.452	99	LEU	0.075	0.001	0.074
168	VAL	0.955	0.977	-0.022	100	ILE	0.528	0.252	0.276
175	PHE	4.614	4.680	-0.066	103	LEU	0.067	0.000	0.067
179	ALA	0.017	0.024	-0.007	104	ILE	0.029	0.000	0.029
183	LYS	2.066	2.660	-0.594	107	ILE	0.012	0.000	0.012
184	MET	2.271	2.887	-0.616	134	PHE	0.005	0.000	0.005
186	ARG	2.467	3.524	-1.057	157	LEU	0.007	0.000	0.007
188	LYS	4.349	4.951	-0.602	160	PHE	0.753	0.728	0.025
189	LYS	3.646	4.828	-1.182	161	MET	0.439	0.378	0.061
192	GLN	0.004	0.115	-0.111	163	VAL	0.164	0.098	0.066
					164	VAL	1.056	0.918	0.138
					167	ILE	0.323	0.298	0.025
					171	ILE	1.492	1.370	0.122
					172	ILE	0.998	0.989	0.009
					174	SER	0.008	0.001	0.007
					180	ILE	0.080	0.004	0.076

Table S3| FEP results for PIP45 to PIP345 in the background of K189A and R80A

Mutation	$PI(4,5)P_2$ to $PI(3,4,5)P_3$ in K189A				$PI(4,5)P_2$ to $PI(3,4,5)P_3$ in R80A			
	$\Delta\Delta G^d$	$\Delta\Delta G_{elec}$	$\Delta\Delta G_{vdW}$	$\Delta\Delta G_{couple}$	$\Delta\Delta G$	$\Delta\Delta G_{elec}$	$\Delta\Delta G_{vdW}$	$\Delta\Delta G_{couple}$
**Total**	**14.958**	**14.424**	**2.121**	**-1.587**	**12.858**	**14.998**	**-0.415**	**-1.726**
**(sderr)**	(1.131)	(1.503)	(0.821)	(0.305)	(2.687)	(3.149)	(0.641)	(0.398)
